# PTEN-induced kinase 1 exerts protective effects in diabetic kidney disease by attenuating mitochondrial dysfunction and necroptosis

**DOI:** 10.7150/ijbs.83906

**Published:** 2023-10-02

**Authors:** Min-Ji Sung, Hyun-Ju An, Min Heui Ha, Seon Hwa Park, Hye Yun Jeong, Jihyun Baek, Sang Ho Lee, Yu Ho Lee, So-Young Lee

**Affiliations:** 1 Devision of Nephrology, Department of Internal Medicine, CHA Bundang Medical Center, CHA University, Seongnam, South Korea.; 2 Devision of Nephrology, Department of Internal Medicine, Kyung Hee University Hospital at Gangdong, Kyung Hee University, Seoul, South Korea.

**Keywords:** PINK1, diabetic kidney disease, diabetic tubulopathy, mitochondria, necroptosis

## Abstract

Mitochondrial dysfunction plays a pivotal role in diabetic kidney disease initiation and progression. PTEN-induced serine/threonine kinase 1 (PINK1) is a core organizer of mitochondrial quality control; however, its function in diabetic kidney disease remains controversial. Here, we aimed to investigate the pathophysiological roles of PINK1 in diabetic tubulopathy, focusing on its effects on mitochondrial homeostasis and tubular cell necroptosis, which is a specialized form of regulated cell death. PINK1-knockout mice showed more severe diabetes-induced tubular injury, interstitial fibrosis, and albuminuria. The expression of profibrotic cytokines significantly increased in the kidneys of diabetic *Pink1^-/-^* mice, which eventually culminated in aggravated interstitial fibrosis. Additionally, the knockdown of PINK1 in HKC-8 cells upregulated the fibrosis-associated proteins, and these effects were rescued by PINK1 overexpression. PINK1 deficiency was also associated with exaggerated hyperglycemia-induced mitochondrial dysfunction and defective mitophagic activity, whereas PINK1 overexpression ameliorated these negative effects and restored mitochondrial homeostasis. Mitochondrial reactive oxygen species triggered tubular cell necroptosis under hyperglycemic conditions, which was aggravated by PINK1 deficiency and improved by its overexpression. In conclusion, PINK1 plays a pivotal role in suppressing mitochondrial dysfunction and tubular cell necroptosis under high glucose conditions and exerts protective effects in diabetic kidney disease.

## Introduction

Mitochondria are energy generators in cells and are critical to cell metabolism, reactive oxygen species (ROS) production, intracellular calcium homeostasis, and cell survival 1. The kidney, which is rich in mitochondria, has a high resting metabolic rate requiring sufficient adenosine triphosphate (ATP) to remove waste and excess fluid from the blood, maintain the acid-base balance, and re-absorb nutrients 2. Recent studies have investigated the role of mitochondria in kidney disease to explore potential therapeutic targets 3.

Necroptosis is a form of programmed cell death mimicking features of apoptosis and necrosis that has attracted attention in various human diseases 4. Necroptotic cell death is initiated when receptor-interacting protein kinase (RIPK) 1 is activated and phosphorylates RIPK3, which triggers phosphorylation of mixed lineage kinase domain-like protein (MLKL) and ultimately leads to plasma membrane disruption and cell lysis 4. Necroptosis has been implicated in the development of acute kidney injury and chronic kidney disease 5-7. The necrosome, which comprises phospho-forms of RIPK1, RIPK3, and MLKL, increases in the proximal tubular cells after acute kidney injury, and drugs inhibiting necroptosis improve cell viability 8-10. Suppression of necroptosis also ameliorated subtotal nephrectomy- and crystal-induced kidney injury models in animal experiments 7,11.

Diabetic kidney disease (DKD) is the leading cause of end-stage kidney disease worldwide. Growing evidence suggests that renal tubular epithelial injuries play an important role in the initiation and progression of DKD 4,12. Among the various factors involved in the pathogenesis of diabetic tubular injury, mitochondrial dysfunction is one the most important components contributing to progressive kidney fibrosis 13. We previously demonstrated impaired mitochondrial biogenesis and dynamics along with reduced ATP production in the renal tubular epithelial cells of diabetic mice. It was also observed that reversing mitochondrial abnormalities could diminish albuminuria and renal fibrosis in diabetic kidneys 14.

PTEN-induced serine/threonine kinase 1 (PINK1) is one of the core organizers of mitochondrial quality control and contributes to mitochondrial homeostasis. We previously demonstrated that peroxisome proliferator-activated receptor gamma coactivator 1-alpha (PGC1α) is involved in mitochondrial dynamics and autophagy in diabetic tubulopathy 15,16. Because *PINK1* is a candidate downstream gene of *PGC1α* 14,17, PINK1 may also play specific roles in the pathogenesis of DKD. Therefore, we aimed to investigate the relationship between PINK1 and tubular cell necroptosis under high glucose conditions and explore its effects on DKD progression.

## Methods

### Animal studies

*Pink1*-knockout mice and their littermates were used in this study. The genotype and phenotype of *Pink1*-knockout mice were confirmed previously 18. Diabetes was induced in 8-week-old male mice by intraperitoneal injection of streptozotocin (STZ; Sigma Aldrich, Burlington, MA) at 50 mg/kg for 5 consecutive days. In the intervention study, four groups of mice were used (n=7 in each group) as follows: (1) normoglycemic *Pink1^+/+^*, (2) normoglycemic *Pink1^-/-^*, (3) diabetic *Pink1^+/+^*, and (4) diabetic *Pink1^-/-^*. All mice were sacrificed 16 weeks after STZ administration, and their kidney tissue samples were collected for analysis. During the experiments, body weights and serum glucose concentrations were measured weekly. All animal experiments were performed in strict accordance with the recommendations of the Guide for the Care and Use of Laboratory Animals of the Korea National Institutes of Health. All animal experiments were performed in compliance with the guidelines of the Animal Research Ethics Committee and the Institutional Animal Care and Use Committee of CHA bio complex (No. 200012).

### Measurements of serum creatinine and urea nitrogen levels

Plasma samples were collected from each mouse at the time of sacrifice. Plasma creatinine levels were measured using a creatinine ELISA kit (Creatinine Assay kit, Abcam, ab65340, Cambridge, UK) following the manufacturer's instructions. Similarly, urea nitrogen levels were assessed using a commercial ELISA kit (Invitrogen, EIABUN, Waltham, MA) according to the manufacturer's instructions.

### Cell culture

The human renal proximal tubular epithelial cell line HKC-8 was obtained from Dr. L. Rausen (Johns Hopkins University, Baltimore, MD) and maintained in Dulbecco's modified Eagle medium supplemented with Ham's F12 medium (DMEM/F12), 5% fetal bovine serum, and 1% penicillin/streptomycin (WelGENE, Daegu, Korea). HKC-8 cells were cultured with 5- or 30-mM D-glucose for 48 hours and were collected for analysis.

Primary renal proximal tubular epithelial cells were isolated from the kidney cortex of 4-week-old *Pink1^+/+^* and* Pink1^-/-^* mice, washed with sterile phosphate buffer saline (PBS), minced, and digested in type 1 collagenase solution at 37°C for 1 hour. After enzyme digestion, cells were washed with PBS, passed through a 100-µm cell strainer (SPL life sciences, Gyeonggi-do, Korea), centrifuged, and resuspended in DMEM/F12 containing 5% fetal bovine serum and 1% penicillin-streptomycin. Cells were placed in 100-mm culture plates and maintained at 37℃ with 5% CO_2._

### Transfection with RNA oligonucleotides

Small interfering RNA (siRNA) duplex oligonucleotides targeting *Pink1* (si*Pink1*; catalog no. 44598) and scrambled negative control siRNA (catalog no. 37007) were purchased from Santa Cruz Biotechnology (Dallas, TX). For functional analyses, cells were transfected with the siRNA oligonucleotides at a final concentration of 60 nM using Lipofectamine RNAiMAX (Gibco-Invitrogen) according to the manufacturer's recommendations.

### Overexpression of PINK1

We used lentiviral expression vector cloned PINK1 human tagged ORF (CAT#: RC206970L4, Origene, Rockville, MD), the pLenti-C-mGFP-P2A lentiviral vector. *Pink1* and control lentiviruses were produced by transfecting Lenti-X 293 T cells (Clontech, Mountain View, CA) with the pLenti-C-mGFP-P2A-Puro-*Pink1*, pLenti-C-mGFP-P2A-Puro (control), and packaging (pCMV-VSV-G, pMDLg/pRRE, and pRSV-Rev; Addgene, Watertown, MA) plasmids using Lipofectamine 3000 (Gibco-Invitrogen). Infectious lentiviruses were harvested 48 hours post-transfection and subsequently used to infect the HKC-8 cells with 4 μg/ml polybrene (Sigma Aldrich).

### Western blot analysis

Cells and kidney tissue samples were washed with PBS and lysed in ice-cold lysis buffer containing a protease inhibitor cocktail (Roche Diagnostics, Mannheim, Germany). Proteins were separated by 8%-15% SDS-PAGE and subsequently transferred into a polyvinylidene difluoride membrane (Merck-Millipore, Madrid, Spain) by electroblotting. The membranes were blocked for 1 hour at 20°C and subsequently incubated overnight at 4°C with anti-PINK1 for mouse (1:200, Abcam, ab23707), anti-α-SMA (alpha-smooth muscle actin, 1:1000, Abcam, ab5694), anti-Fibronectin (1:2000, Abcam, ab2413), anti-TGF-β1 (transforming growth factor-beta 1, 1:1000, Abcam, ab215715), anti-TNFα (tumor necrosis factor-alpha 1, Gibco-Invitrogen, PA1-40281), anti-phospho-MLKL (1:1000, Cell Signaling Technology, #37333, Danvers, MA), anti-phospho-RIPK3 (1:1000, Cell Signaling Technology, #91702), anti-phospho-RIPK1 (S161, 1:1000, Gibco-Invitrogen, PA5-105640), anti-phospho-RIPK1 (S166, 1:1000, Cell Signaling Technology, #44590), anti-MLKL (1:1000, Cell Signaling Technology, #37705), anti-RIPK1 (1:1000, Santa Cruz Biotechnology, sc-133102), anti-RIPK3 (1:1000, Santa Cruz Biotechnology, sc-374639), anti-E-cadherin (1:1000, Santa Cruz Biotechnology, sc-8426), and anti-PINK1 (1:500, Santa Cruz Biotechnology, sc-517353) for human primary antibodies. Subsequently, the membranes were stained with horseradish peroxidase (HRP)-conjugated goat anti-rabbit or -mouse immunoglobulin G (1:5000, Enzo Life Sciences Inc., ADI-SAB-300-J, Farmingdale, NY). Immunoreactive bands were detected using chemiluminescence (enhanced chemiluminescence; Bio-Rad Laboratories, Hercules, CA). Glyceraldehyde 3-phosphate dehydrogenase (GAPDH) (1:3000, Santa Cruz Biotechnology, sc-47724) and β-actin (1:2000, Santa Cruz Biotechnology, sc-47778) were used as the internal controls.

### Isolation of RNA and real-time quantitative PCR

Total RNA was isolated from cultured cells or frozen kidney tissues using TRIzol® reagent (Gibco-Invitrogen) or the RNeasy Mini kit (Qiagen, Venlo, Limburg, Netherlands), respectively, according to the manufacturer's protocols. To quantify mRNA expression, total RNA (1 µg) was reverse transcribed into cDNA using an AMPIGENE® cDNA Synthesis kit (Enzo Life Sciences Inc.). The reaction mixtures were incubated sequentially at 42°C for 30 minutes and 85°C for 10 minutes. Real-time quantitative PCR (qPCR) for mRNA determination was performed using Fast Start Universal SYBR Green Master (Enzo Life Sciences Inc.) and an iCycler real-time PCR detection system (Bio-Rad Laboratories) according to the manufacturer's protocols. The qPCR reaction conditions were as follows: denaturation at 95°C for 2 minutes, followed by 40 cycles of 95°C for 5 seconds, 60°C for 30 seconds, and 72°C for 30 seconds. The sequences of specific primers used for qPCR analyses of mRNA are listed in **Table S1**. The mRNA expression levels were calculated using the 2^-ΔΔCq^ method.

### Renal histopathology and immunohistochemical staining

Paraffin-embedded sections of mouse kidney specimens were prepared and used for periodic acid-Schiff and Masson trichrome staining. The semiquantitative assessment of tubular injury and interstitial fibrosis was performed in a blinded manner, and the specific scoring system used in these evaluations has been previously described 19. In brief, assessment of tubular injury involved grading from 0 to 3 based on the extent of tubular dilatation and epithelial cell destruction: 0 for absence of injury, 1 for 1-25% affected, 2 for 26-50% affected, and 3 for >50% affected. Interstitial fibrosis was quantified by calculating the percentage of positive fibrotic area in Masson-Trichrome-stained tissues.

Immunohistochemistry of kidney tissue samples was performed using a ready-to-use IHC/ICC kit (BioVision, Milpitas, CA) according to the manufacturer's protocol. Briefly, mouse kidney tissue samples were fixed with 4% formaldehyde at 20°C for 24 hours. Next, the paraffin-embedded tissues were cut into 4-µm‑thick sections, deparaffinized, rehydrated, and microwaved in citrate buffer (Abcam, ab93678) and subsequently used for antigen retrieval. The slides were incubated in 3% H_2_O_2_ at 20°C for 30 minutes to quench the endogenous peroxidase activity, and subsequently, blocked-in blocking buffer (BioVision) at 20°C for 15 minutes, followed by incubation with 8-hydroxy-2'-deoxyguanosine (8-OHdG, 1:2000, Santa Cruz Biotechnology, sc-66036), α-SMA (1:500, Cell Signaling Technology, ab5694), phospho-RIPK3 (1:200, Cell Signaling Technology, #91702), phospho-MLKL (1:100, Cell Signaling Technology, #37333), TNFα (1:100, Abcam, PA1-40281), and fibronectin (1:100, Abcam, ab2413) antibodies at 20°C for 30 minutes. After incubation with HRP-anti-mouse or -rabbit IgG polymer at 20°C for 20 minutes and washing with PBS, the tissue sections were treated with 3,3'-diaminobenzidine at 20°C for 10 minutes, followed by counterstaining with hematoxylin at 20°C for 1 minute. Images were captured using an ECLIPSE Ts2 microscope (magnification ×200; Nikon Instruments Inc., Melville, NY). Immunohistochemistry quantification was performed using pixel density analysis in Photoshop. After immunostaining, all tissue slides were scanned using a slide scanner, and 10 sections per a slide were selected at 200x magnification. The stained area's color was identified, and the number of pixels was measured with a fixed color range to ensure consistency. The evaluation entailed determining the proportion of stained areas relative to the total number of image pixels.

### Measurement of mitophagic activity

Mitochondria targeted Keima-Red-Mito-7 (mt-Keima, Addgene, plasmid #56018) were transfected using Lipofectamine 3000 according to the manufacturer's protocol. After incubation in the plasmid solution for 24 hours, the cells were incubated with DMEM/F12 under 5- or 30-mM D-glucose for an additional 48 hours. The cells were counterstained with 4′,6-diamidino-2-phenylindole (DAPI) to delineate the nuclei and examined through confocal microscopy (LSM-700; Carl Zeiss, Jena, Germany). Photoshop program was utilized to select the fluorescence color of each image pixel. A fixed range value for this color was established and consistently maintained across all experimental groups. The number of selected pixels was then measured, allowing for an accurate assessment of mt-Keima levels in the studied samples. Mitophagic activity was expressed as ratios between the measured values of 458nm (green) and 561nm (red) fluorescence.

### Measurement of reactive oxygen species

To assess intracellular and mitochondrial reactive oxygen species (ROS) production, cells were incubated with dihydroethidium (DHE) (15 µM, Life Technologies, Seoul, Korea) and MitoSOX (5 µM, Life Technologies) at 37°C for 30 minutes and examined using confocal microscopy (LSM-700; Carl Zeiss). DHE was excited at 518 nm, and the fluorescence emission at 610 nm was measured. In contrast, MitoSOX Red was excited at 510 nm, and the fluorescence emission at 580 nm was measured.

### Measurement of mitochondrial membrane potential

The mitochondrial inner membrane electrochemical potential **(**ΔΨm) was assessed using JC-1 dye according to the manufacturer's instructions (mitochondrial membrane potential assay kit, #ab113850, Abcam). JC-1 is a lipophilic fluorescent cation that emits green and red fluorescence at low ΔΨm and high ΔΨm, respectively. HKC-8 cells were collected and stained with 10 µg/mL JC-1 at 37°C in the dark for 15 minutes, followed by measurement of the absorbance at 590 nm (aggregate emission) and 530 nm (monomer species) in a microplate reader (Molecular Devices, Sunnyvale, CA).

### Mutant mitochondrial DNA detection

Genomic DNA was isolated to screen for the 4,977-bp and 3,860-bp deletions in human mitochondrial DNA (mtDNA) and mouse mtDNA, respectively. The primers used for mtDNA detection are provided in **Table S1**. qPCR conditions for human mtDNA were as follows: pre-denaturation at 94°C for 5 minutes; 30 cycles at 94°C for 10 seconds, 58°C for 45 seconds, and 7°C for 50 seconds; and a final extension at 72°C for 10 minutes. qPCR conditions for mouse mtDNA were as follows: pre-denaturation at 94°C for 5 minutes; 35 cycles at 94°C for 15 seconds, 59°C for 30 seconds, and 72°C for 50 seconds; and a final extension at 72°C for 10 minutes. To quantify the mtDNA/genomic DNA ratio, qPCR was used to amplify one gene from the mitochondrial genome (COX1) and one from the nuclear genome (nuclear β-actin).

### Electron microscopy

After the removal of the kidneys, the tissues were immediately diced into 1-mm^3^ pieces and fixed with 2.5% glutaraldehyde and 2% paraformaldehyde in sodium cacodylate buffer (pH 7.2) at 4°C. Subsequently, tissue specimens were post-fixed in 1% osmium tetroxide (OsO_4_) containing 1.5% potassium ferrocyanide for 30 minutes at 4°C. Kidney tissue samples were cut into semi-thin (1 μm) and ultra-thin sections (80 nm) and stained with 1% uranyl acetate and lead citrate. The ultrastructure of the cells and mitochondria was analyzed using transmission electron microscopy (JEM-1230, Jeol, Japan). Mitochondrial area and length in renal proximal tubular cells were quantified by averaging ten random electron microscopic sections.

### Seahorse assays

Cellular and mitochondrial respiration was quantified using the Seahorse Extracellular Flux Analyzer XF-^e^24 (Seahorse Agilent Technologies) and the commercial XF Cell Mito Stress Test kit (Agilent Technologies, Santa Clara, CA), respectively. Each 6-well Seahorse cell culture plate was precoated with rat tail collagen type I (Sigma). Naive and differentiated HKC-8 cells were detached using 0.05% trypsin-EDTA, counted, and plated at 3,000 cells/well density. Cells were allowed to attach for 16-20 hours. The initial incubation medium was replaced with Seahorse XF base DMEM medium, and the plate was transferred into the XF-^e^24 analyzer for calibration and measurement of the oxygen consumption rate (OCR). The duration of each step within a measurement cycle was adjusted to 3 minutes of mixing and 3 minutes of measurement time. Respiration of cells was quantified as OCR at baseline and after treatment of each well with appropriate mitochondrial modulators, such as oligomycin (1.5 μM), FCCP (0.5 μM), and rotenone and antimycin A (0.5 μM).

### Statistics

Data are expressed as mean ± standard errors. An independent t-test was used to compare the differences between the two groups, and a one-way analysis of variance with a post hoc Tukey's test was performed to compare the differences between more than two groups. *p*-value less than 0.05 was considered as statistically significant.

## Results

### PINK1 deficiency aggravates kidney dysfunction, albuminuria, and renal morphological changes and in STZ-induced diabetic mice

**Figure 1:**
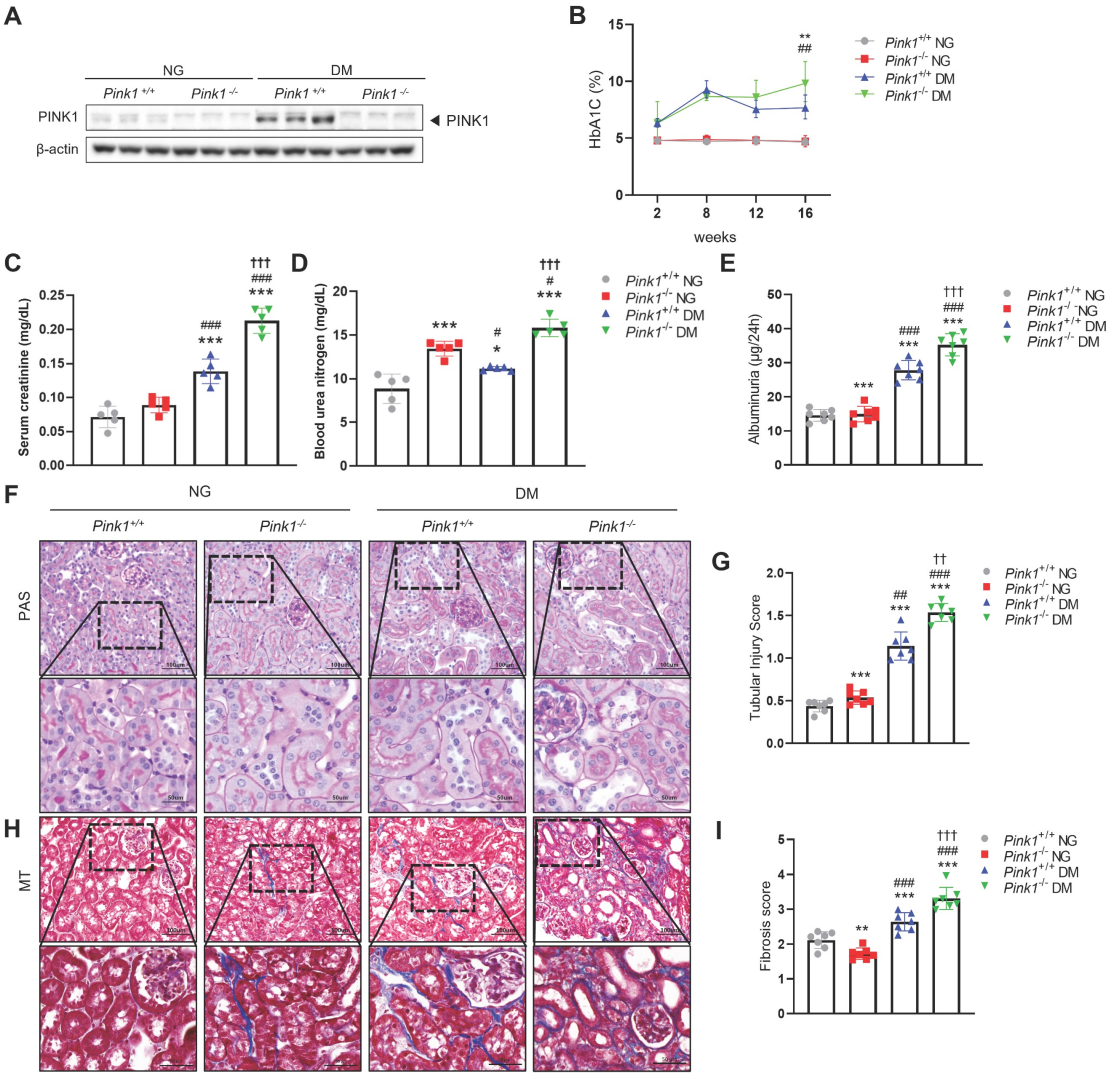
** PINK1 deficiency aggravates kidney dysfunction, albuminuria, and renal morphological changes in the kidneys of diabetic mice. (A)** Western blotting of PINK1 in whole kidney lysate obtained from *Pink1*^+/+^ and *Pink1*^-/-^ mice with normal or high blood glucose levels. (**B**) % of HbA1c during a 16-week period. **(C** and **D)** Levels of serum creatinine (**C**) and blood urea nitrogen (**D**) across groups. (**E**) Quantification of 24-hour albuminuria. (**F**) Representative photomicrographs of periodic acid-Schiff-stained kidneys of *Pink1*^+/+^ and *Pink1*^-/-^ mice with normal or high serum glucose levels. (**G**) A semiquantitative assessment of the tubular injury score was performed. (**H**) Representative photomicrographs of Masson trichrome-stained kidneys of *Pink1*^+/+^ and *Pink1*^-/-^ mice with normal or high serum glucose levels. (**I**) A semiquantitative assessment of renal fibrosis was performed. Scale bar: (**F** and** H**) non-magnified images (upper panels) = 100 μm, magnified images (lower panels) = 50 μm. Values are expressed as mean ± standard error. n = 7 per group, ^*^p < 0.05, ^**^p < 0.01, ^***^p < 0.001 vs *Pink1*^+/+^ NG, ^#^*p* < 0.05, ^##^*p* < 0.01, ^###^*p* < 0.001 vs *Pink1*^-/-^ NG, ^†^*p* < 0.05, ^††^*p* < 0.01 vs *Pink1*^+/+^ DM. **Abbreviations**: PINK1, PTEN-induced serine/threonine kinase 1; HbA1c, glycated hemoglobin; NG, normoglycemia; DM, diabetes mellitus; PAS, periodic acid-Schiff; MT, Masson trichrome.

To determine the effect of PINK1 deficiency on DKD *in vivo*, we first induced hyperglycemia in *Pink1^+/+^* and *Pink1^-/-^* mice through STZ injection. PINK1 deficiency in *Pink1^-/-^* mice was confirmed by western blotting (**Figure 1A**). Diabetes induced an increase in PINK1 expression in the kidneys. Both *Pink1^+/+^* and *Pink1^-/-^* diabetic mice had higher glycated hemoglobin (HbA1c) levels than mice with normal blood glucose, and no significant difference was observed in the HbA1c levels between *Pink1^+/+^* and *Pink1^-/-^* mice (**Figure 1B**). In diabetic mice, serum creatinine and urea nitrogen levels were significantly increased compared to control mice, and the absence of PINK1 further exacerbated diabetes-induced kidney dysfunction (**Figure 1C and 1D**). Albuminuria significantly increased in diabetic *Pink1^-/-^* mice compared with that in diabetic *Pink1^+/+^* mice (**Figure 1E**). Subsequently, we examined the loss of PINK1 in developing tubular injury and interstitial fibrosis using periodic acid-Schiff and Masson trichrome stain. Histological analyses showed tubular dilatation and epithelial disruption in the diabetic mice group. Notably, the tubular injury was most prominent in the kidneys of diabetic *Pink1^-/-^*mice (**Figure 1F and 1G**). Diabetic mice demonstrated a marked increase in renal interstitial collagen deposition, and loss of PINK1 significantly aggravated interstitial fibrosis (**Figure 1H and 1I**).

These results suggest that PINK1 plays an important role in tubulointerstitial injury and fibrosis in DKD.

### Loss of PINK1 promotes profibrotic phenotypes of renal tubular epithelial cells in diabetic mice

**Figure 2:**
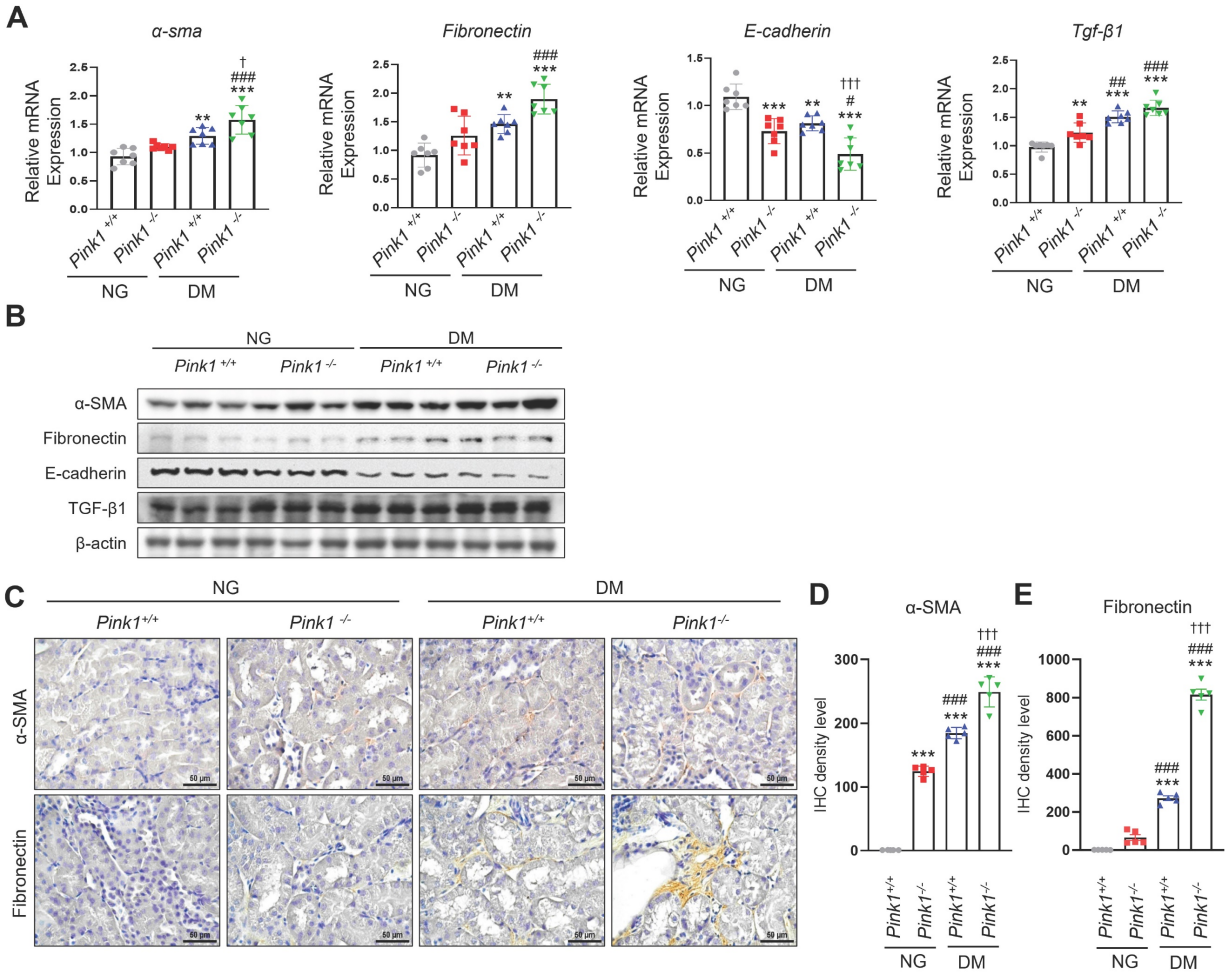
** PINK1 deficiency increases the expression of profibrotic molecules in the kidneys of diabetic mice.** (**A** and** B**) mRNA levels (**A**) and western blotting (**B**) of α-SMA, fibronectin, E-cadherin, and TGF-β1 in the kidneys of *Pink1*^+/+^ and *Pink1*^-/-^ mice with normal or high serum glucose levels. (**C**) Representative immunohistochemical staining of α-SMA and fibronectin in the kidneys of *Pink1*^+/+^ and *Pink1*^-/-^ mice with normal or high serum glucose levels. (**D** and** E**) A semiquantitative assessment of the staining intensity in the renal tubules was performed. Quantitative analysis of **Figure 2B** are shown in **Figure S1**. (**C**) Scale bar = 100 μm. Data are representative of three independent experiments and is expressed as means ± standard error. n = 7 per group, ^*^p < 0.05, ^**^p < 0.01, ^***^p < 0.001 vs *Pink1*^+/+^ NG, ^#^*p* < 0.05, ^##^*p* < 0.01 vs *Pink1*^-/-^ NG, ^†^*p* < 0.05 vs *Pink1*^+/+^ DM. **Abbreviations**: PINK1, PTEN-induced serine/threonine kinase 1; α-SMA, alpha-smooth muscle actin; TGF-β1, transforming growth factor-β1; NG, normoglycemic; DM, diabetes mellitus; IHC, immunohistochemistry.

After kidney injury, renal tubular epithelial cells produce profibrotic cytokines, contributing to renal fibrogenesis 20. We observed that α-SMA and fibronectin mRNA and protein expression, which are profibrotic markers, significantly increased, whereas E-cadherin levels, which are epithelial markers, decreased in the kidneys of PINK1-deficient diabetic mice compared with PINK1-wild-type diabetic mice (**Figure 2A, 2B, and Figure S1**). TGF-β1 expression, which is a critical mediator of kidney fibrosis released by damaged renal epithelial cells 21, was also increased in the kidneys of PINK1-deficient mice. Immunohistochemical analysis showed that the expression of α-SMA and fibronectin increased in diabetic kidneys from PINK1-deficient mice (**Figure 2C-E**).

These results suggest that renal tubular epithelial cells acquire profibrotic phenotypes under hyperglycemic conditions, and PINK1 deficiency further accelerates these effects.

### PINK1 regulates renal tubular epithelial phenotypes under high glucose conditions *in vitro*

**Figure 3:**
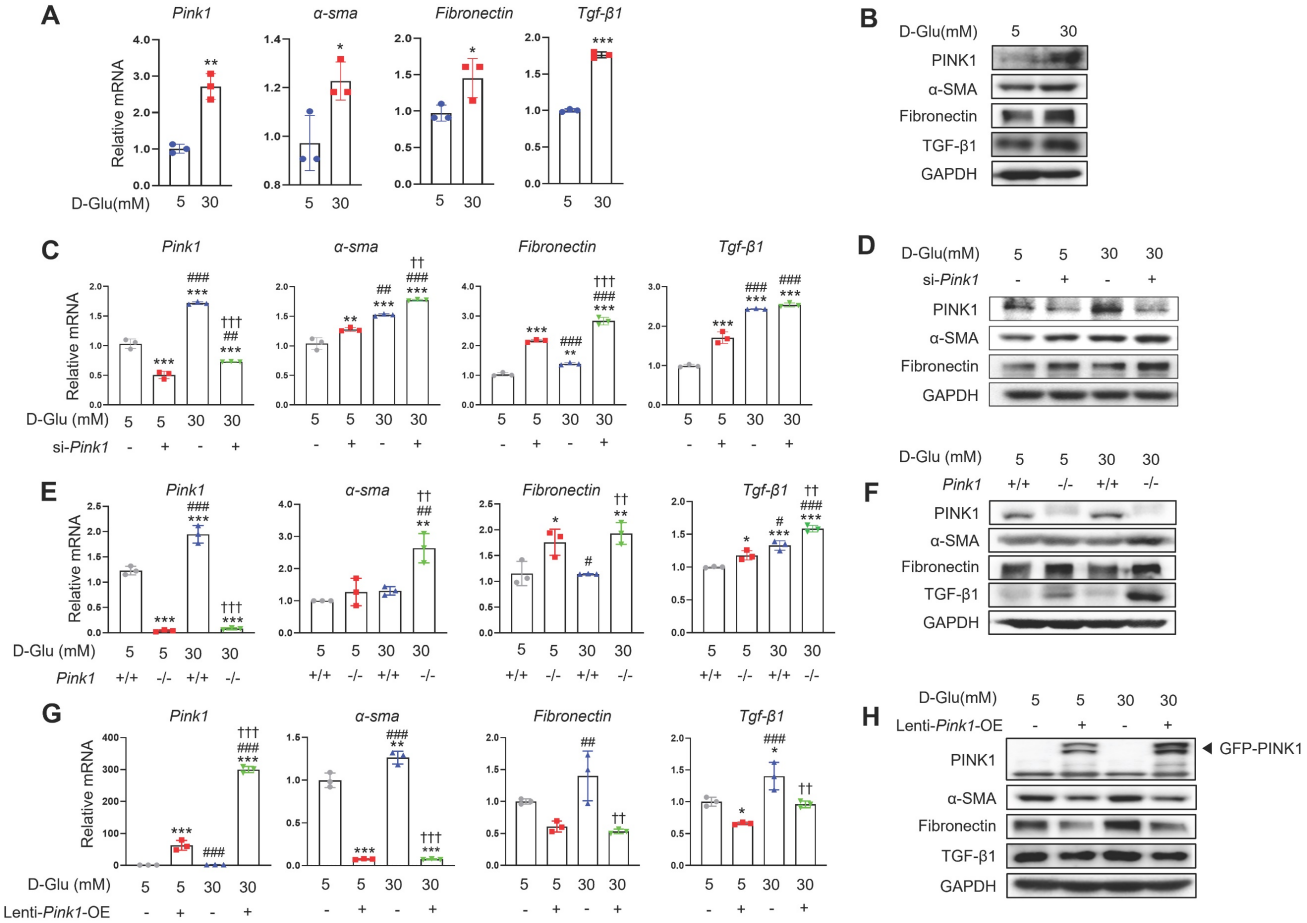
** PINK1 regulates the profibrotic phenotypes of renal tubular epithelial cells under high glucose conditions. (A)** mRNA levels of *Pink1*, α-SMA, fibronectin, and TGF-β1 in human renal proximal tubular cells (HKC-8) after treatment with 5- or 30-mM D-glucose. **(B)** Western blotting of PINK1, α-SMA, fibronectin, and TGF-β1 in HKC-8 cell lysates after treatment with 5- or 30-mM D-glucose. **(C)** Quantification of the expression of *Pink1*, α-SMA, fibronectin, and TGF-β1 mRNAs in HKC-8 cells with control siRNA or *Pink1* siRNA under 5- or 30-mM D-glucose. **(D)** Western blotting of PINK1, α-SMA, and fibronectin in control or PINK1 siRNA-treated HKC-8 cells under 5- or 30-mM D-glucose. **(E)** Quantification of the expression of *Pink1,* α-SMA, fibronectin, and TGF-β1 mRNA in primary renal tubular epithelial cells of *Pink1*^+/+^ and *Pink1*^-/-^ mice after treatment with 5- or 30-mM D-glucose. **(F)** Western blotting of α-SMA, fibronectin, and TGF-β1 in 5- or 30-mM D-glucose-treated primary renal tubular epithelial cells obtained from *Pink1*^+/+^ or *Pink1*^-/-^ mice. **(G)** Quantification of the expression of *Pink1*, α-SMA, fibronectin, and TGF-β1 mRNA in control or GFP-*Pink1* infected HKC-8 cells after treatment with 5- or 30-mM D-glucose. **(H)** Western blotting of α-SMA, fibronectin, and TGF-β1 in PINK1-overexpressed HKC-8 cells after treatment with 5- or 30-mM D-glucose. Quantitative analysis of **Figure 3B, 3D, 3F, and 3H** are shown in **Figure S2**. Shown are representatives of three independent experiments and are expressed as means ± standard error. ^*^p < 0.05,^ **^p < 0.01,^ ***^p < 0.001 vs *Pink1^+/+^* NG, ^#^*p* < 0.05,^ ##^*p* < 0.01 vs *Pink1^-/-^* NG, **^††^***p* < 0.01, **^†††^***p* < 0.001 vs *Pink1^+/+^* DM. **Abbreviations:** PINK1, PTEN-induced serine/threonine kinase; α-SMA, alpha-smooth muscle actin; TGF-β1, transforming growth factor-β1; NG, normal glucose; DM, diabetes mellitus; GAPDH, glyceraldehyde 3-phosphate dehydrogenase; OE, overexpression; GFP, green fluorescent protein.

Next, we explored the pathophysiologic roles of PINK1 in diabetic tubulopathy using HKC-8 cells. Hyperglycemia upregulated the expression of PINK1 and profibrotic markers in these cells, which is consistent with our *in vivo* data (**Figure 3A, 3B, and Figure S2A**). To investigate whether PINK1 could modulate phenotypic alterations of renal tubular epithelial cells under high glucose conditions, we constructed *Pink1* knockdown cells with *Pink1*-siRNA transfection and *Pink1* overexpression cells with lentiviral vectors. *Pink1* siRNA significantly enhanced the profibrotic phenotype in renal proximal tubular epithelial cells in high glucose media (**Figure 3C, 3D, and Figure S2B**). These effects were consistently observed in primary cultured renal tubular epithelial cells obtained from *Pink1^+/+^* and *Pink1^-/-^* mice (**Figure 3E, 3F, and Figure S2C**). In contrast, *Pink1* overexpression significantly attenuated hyperglycemia-induced phenotypic changes in HKC-8 cells (**Figure 3G, 3H, and Figure S2D**).

These data suggest that the PINK1 level is important in regulating renal tubular epithelial phenotypes in diabetic environments.

### PINK1 regulates mitochondrial integrity and mitophagy in renal tubular epithelial cells

**Figure 4:**
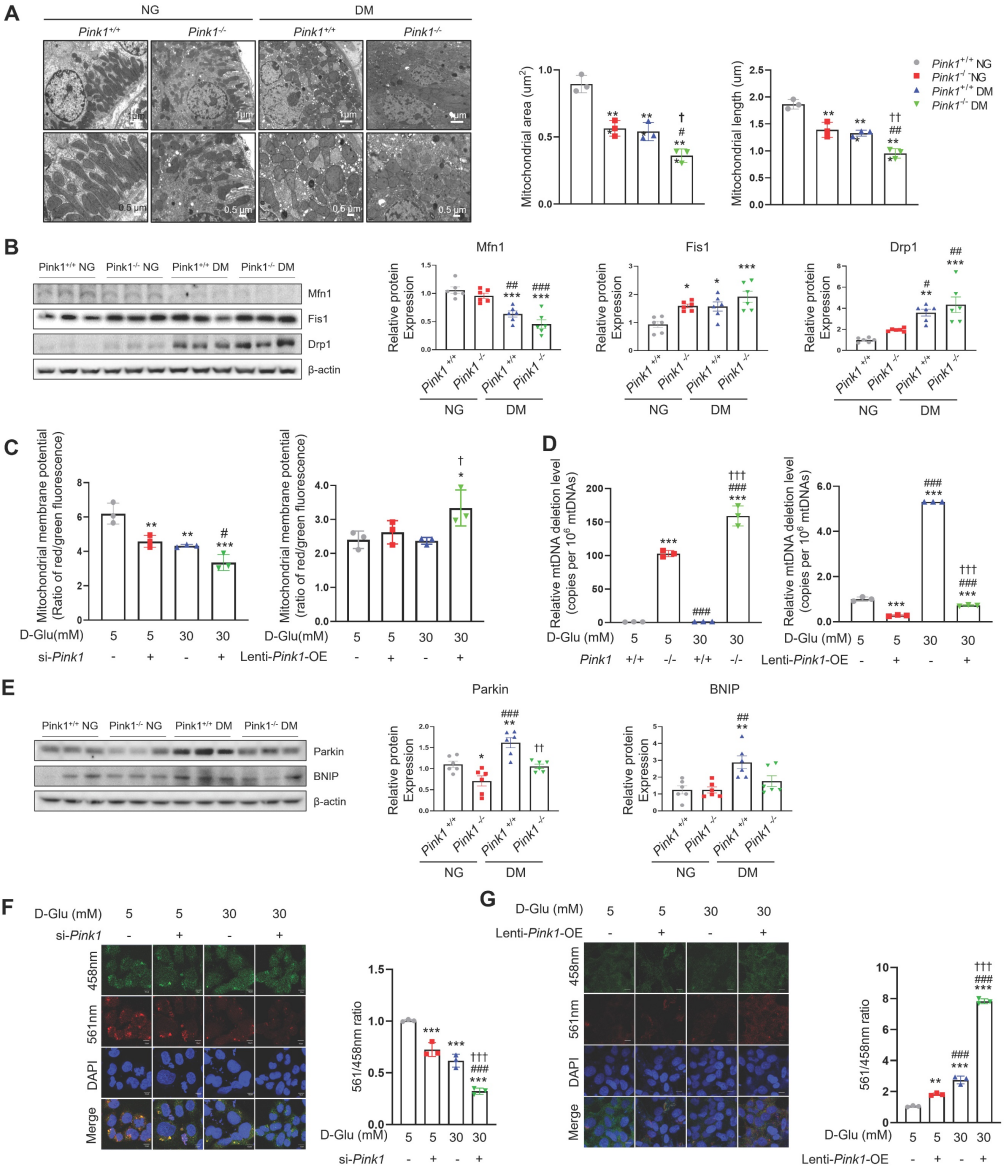
** PINK1 maintain mitochondrial homeostasis and mitophagic function in renal tubular epithelial cells under high glucose conditions. (A)** Representative electron microscopy images and the quantification of mitochondrial area and length in renal proximal tubular epithelial cells of *Pink1^+/+^* and *Pink1^-/-^* mice with normal or high serum glucose levels. **(B)** Western blotting of mitofusin 1 (Mfn1), mitochondrial fission 1 (Fis1), and dynamin-related protein 1 (Drp1) in the kidneys of *Pink1*^+/+^ and *Pink1*^-/-^ mice with normal or high serum glucose levels, and their quantification. **(C)** The mitochondrial membrane potential of HKC-8 cells with *Pink1* siRNA and GFP-*Pink1* infected HKC-8 cells after treatment with 5- or 30-mM D-glucose. **(D)** The mouse mitochondrial DNA 3,860-bp deletion in primary cultures of kidney proximal tubule cells obtained from *Pink1^+/+^* or *Pink1^-/-^* mice and the human mitochondrial DNA 4,977-bp deletion in control or GFP-*Pink1* infected HKC-8 cells after treatment with 5- or 30-mM D-glucose. **(E)** Western blotting of Parkin and Bcl-2/adenovirus E1B 19 kD-interacting protein 3 (BNIP3) in the kidneys of *Pink1*^+/+^ and *Pink1*^-/-^ mice with normal or high serum glucose levels, and their quantification. **(F)** Representative fluorescence images of the mt-Keima and their quantification in HKC-8 cells with control or *Pink1* siRNA after treatment with 5- or 30-mM D-glucose. **(G)** Representative fluorescence images of the mt-Keima and their quantification in control or GFP-*Pink1* infected HKC-8 cells after treatment with 5- or 30-mM D-glucose. Scale bar: (**A**) 1 μm and 0.5 μm in upper and lower images, respectively, (**F** and** G**) 10 μm. (**A, B, and E**) n = 6 per group. **(C, D, F, and G)** Data are representative of three independent experiments and are expressed as means ± standard error. n = 3 per group. ^**^p < 0.01,^ ***^p < 0.001 vs *Pink1*^+/+^ NG, ^#^*p* < 0.05,^ ##^*p* < 0.01,^ ###^*p* < 0.001 vs *Pink1*^-/-^, NG, **^†^***p* < 0.05, **^††^***p* < 0.01, **^†††^***p* < 0.001 vs *Pink1*^+/+^ DM. **Abbreviations:** PINK1, PTEN-induced serine/threonine kinase 1; NG, normal glucose; DM, diabetes mellitus; Mfn, mitofusin 1; Fis1, mitochondrial fission 1; Drp1, dynamin-related protein 1; OE, overexpression; mtDNA, mitochondrial DNA; BNIP, Bcl-2/adenovirus E1B 19 kD-interacting protein 3; mt-Keima, mitochondria-targeted Keima.

Mitochondria are dynamic organelles that continuously fuse and divide to maintain their networks. Mitochondrial fragmentation is linked with mitochondrial dysfunction, including loss of mitochondrial membrane potential, impaired respiration, oxidative phosphorylation, and increased mitochondrial ROS formation 19. PINK1 deficiency accelerated hyperglycemia-induced mitochondrial fission in the renal tubular cells of diabetic mice (**Figure 4A**). Western blot analysis consistently revealed that sustained hyperglycemia resulted in reduced expression of mitofusin 1 (Mfn1), an indicator of mitochondrial fusion (**Figure 4B**). Additionally, there was an increase in the expression of mitochondrial fission 1 (Fis1) and dynamin-related protein 1 (Drp1), markers of mitochondrial fission. Moreover, these alterations were exacerbated by loss of PINK1. The siRNA for *Pink1* significantly reduced mitochondrial membrane potential, whereas *Pink1* overexpression restored it in HKC-8 cells under high glucose conditions (**Figure 4C**). We assessed the frequency of mitochondrial DNA deletion (D17), which is coupled with damaged mitochondria 19,22.* Pink1* downregulation caused an increased mouse mtDNA 3,860-bp deletion in primary cultures of kidney proximal tubule cells, whereas *Pink1* upregulation was associated with less frequent human mtDNA 4,977-bp deletion in renal tubular epithelial cells under hyperglycemic conditions (**Figure 4D**). Next, we examined the associations between PINK1 and mitophagy, a cellular process to remove dysfunctional mitochondria via the autophagic machinery 23. Western blot analysis demonstrated an elevation in the expressions of Parkin and Bcl-2/adenovirus E1B 19 kD-interacting protein 3 (BNIP3), both indicative of mitophagy, in the kidneys of diabetic mice (**Figure 4E**). However, their expressions were significantly reduced by loss of PINK1. We also used the mt-Keima probe to detect functional mitophagy as indicated by red puncta. Mitophagic function was suppressed under high glucose conditions, and *Pink1* knockdown further suppressed it (**Figure 4F**). In contrast, hyperglycemia-induced mitophagic dysfunction was successfully rescued by *Pink1* overexpression (**Figure 4G**).

### PINK1 is involved in mitochondrial electron transport chain complex expressions and cellular oxygen consumption

**Figure 5:**
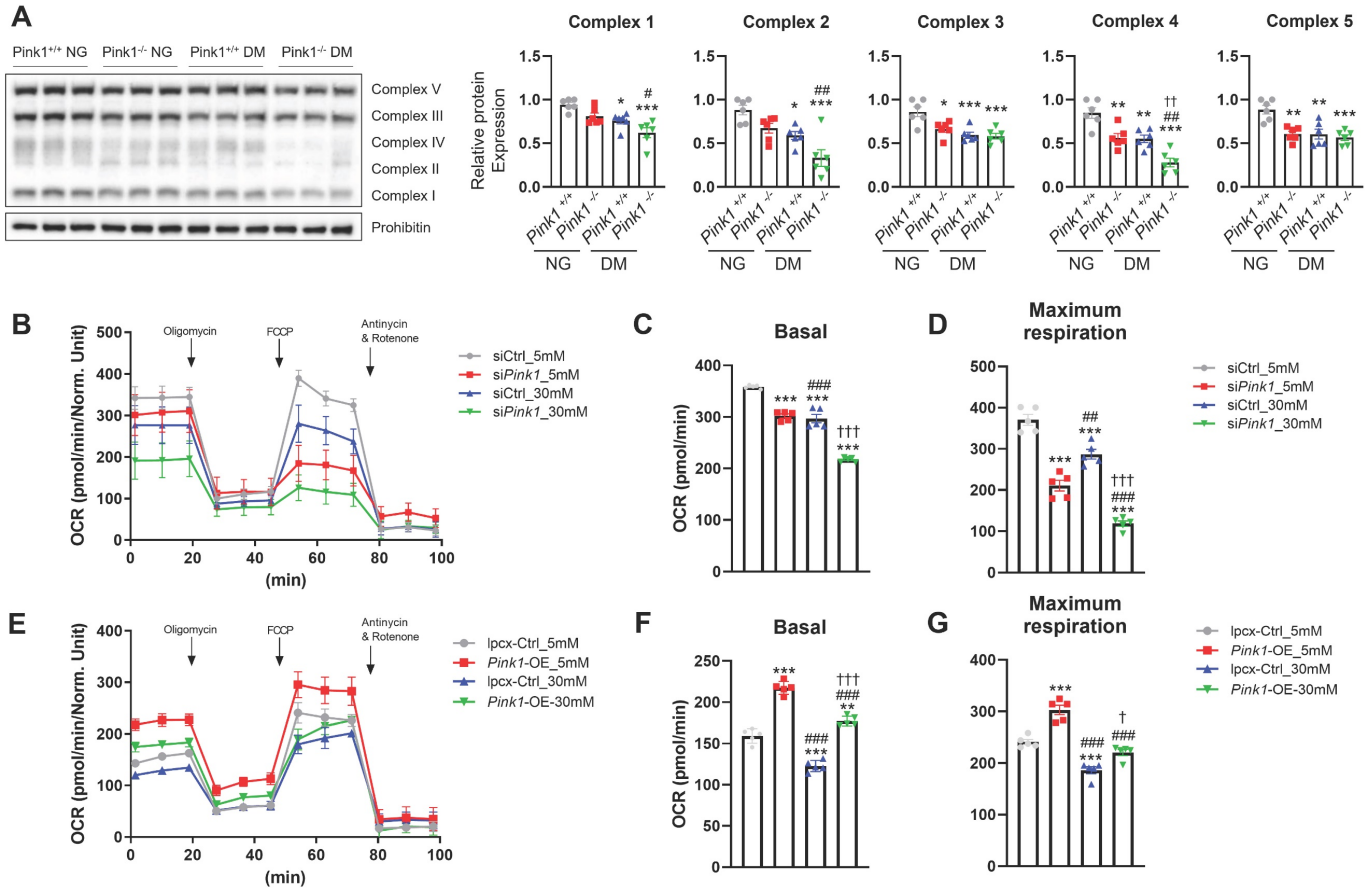
** PINK1 is involved in mitochondrial electron transport chain complex expressions and cellular oxygen consumption. (A)** Western blotting of electron transport chain complex I-V in the kidneys of *Pink1^+/+^* and* Pink1^-/-^* mice with normal or high serum glucose levels. **(B-D)** Representative Seahorse assay traces of HKC-8 cells with control or Pink1 siRNA after treatment with 5- or 30-mM D-glucose and the quantification of OCR at basal and maximal respiration. **(E-G)** Representative Seahorse assay traces of control or GFP-Pink1 infected HKC-8 cells after treatment with 5- or 30-mM D-glucose and the quantification of OCR at basal and maximal respiration. (**A**) n = 6 per group, **(B-G)** Data are representative of five independent experiments and are expressed as means ± standard error. ^**^p < 0.01,^ ***^p < 0.001 vs *Pink1*^+/+^ NG, ^#^*p* < 0.05,^ ##^*p* < 0.01,^ ###^*p* < 0.001 vs *Pink1*^-/-^, NG, **^†^***p* < 0.05, **^††^***p* < 0.01, **^†††^***p* < 0.001 vs *Pink1*^+/+^ DM. **Abbreviations**: PINK1, PTEN-induced serine/threonine kinase 1; NG, normal glucose; DM, diabetes mellitus; OCR, oxygen consumption rate; OE, overexpression.

The electron transport chain (ETC) is a cascade of electron transporters that play a pivotal role in generating large number of ATPs and also serve as a major source of mitochondrial ROS within the renal proximal tubular cells 24. Western blot analysis of whole kidney lysates showed that demonstrated that diabetes induced significant reductions in the expressions of all the mitochondrial ETC complexes (**Figure 5A**). Moreover, the absence of PINK1 resulted in additional decreases in the expressions of ETC complex II and IV, while complexes I, III, and V remained unaffected. Seahorse assay showed that *Pink1* deficiency aggravated hyperglycemia-induced decreases in basal and maximal oxygen consumption rates, which are indicators of mitochondrial dysfunction (**Figure 5B-D**). The hyperglycemia-induced mitochondrial stress was ameliorated by *Pink1* overexpression (**Figure 5E-G**).

### PINK1 controls the formation of reactive oxygen species in renal tubular epithelial cells

**Figure 6:**
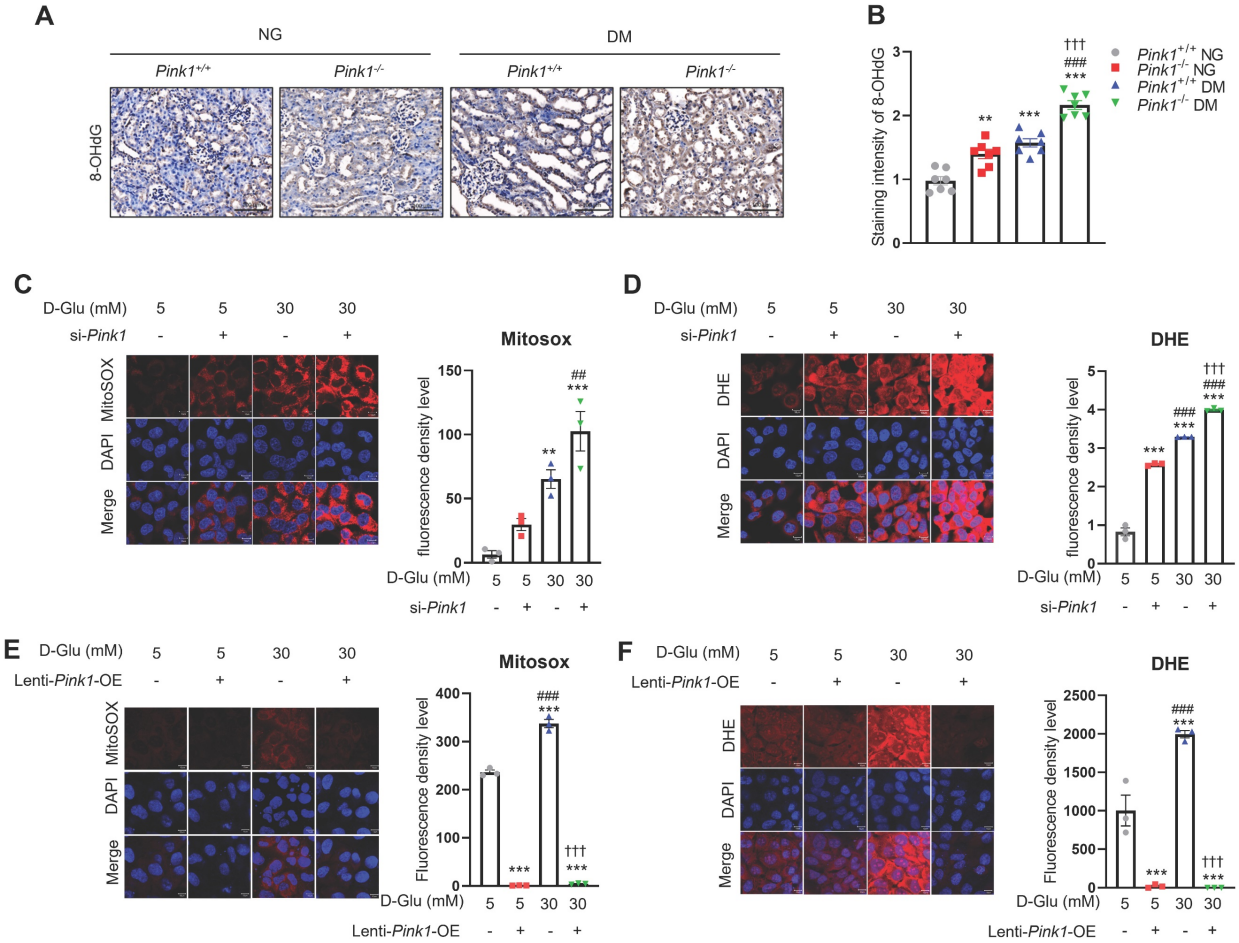
** PINK1 regulates the generation of reactive oxygen species in renal tubular epithelial cells under high glucose conditions. (A** and** B)** Representative immunohistochemical staining of 8-OHdG in the kidneys of *Pink1^+/+^* and* Pink1^-/-^* mice with normal or high serum glucose levels. A semiquantitative assessment of the staining intensity in the renal tubules was performed. **(C)** Representative images of MitoSOX red staining of superoxide radicals and their quantification in HKC-8 cells with control siRNA or *Pink1* siRNA after treatment with 5- or 30-mM D-glucose. **(D)** Immunofluorescence images showing DHE staining and their quantification in HKC-8 cells with control or *Pink1* siRNA after treatment with 5- or 30-mM D-glucose. **(E)** Representative images of MitoSOX red staining of superoxide radicals and their quantification in control or GFP-*Pink1* infected HKC-8 cells after treatment with 5- or 30-mM D-glucose. **(F)** Immunofluorescence images of DHE staining and their quantification in control or GFP-*Pink1* infected HKC-8 cells after treatment with 5- or 30-mM D-glucose. Scale bar: (**A**) 100 μm, (**C-F**) 100 μm. (**A**) n = 7 per group, (**C-F**) Data is representative of three independent experiments and expressed as means ± standard error. n = 3 per group. ^**^p < 0.01,^ ***^p < 0.001 vs *Pink1*^+/+^ NG, ^#^*p* < 0.05,^ ##^*p* < 0.01,^ ###^*p* < 0.001 vs *Pink1*^-/-^, NG, **^†^***p* < 0.05, **^††^***p* < 0.01, **^†††^***p* < 0.001 vs *Pink1*^+/+^ DM. **Abbreviations:** PINK1, PTEN-induced serine/threonine kinase 1; NG, normal glucose; DM, diabetes mellitus; 8-OHdG, 8-hydroxy-2′-deoxyguanosine; DHE, dihydroethidium; OE, overexpression.

Sustained hyperglycemic stimuli are known to increase ROS generation in the renal tubules 25. Immunofluorescence staining showed a significant increase in 8-OHdG, which is an indicator of oxidative stress, in renal tubular cells of diabetic mice (**Figure 6A and 6B**). Moreover, PINK1 deficiency worsened diabetes-induced intrarenal ROS formation. Consistently, *in vitro* experiments demonstrated that *Pink1* depletion augmented hyperglycemia-induced overproduction of mitochondrial and cellular ROS (**Figure 6C and 6D**), whereas its overexpression ameliorated ROS generation in HKC-8 cells (**Figure 6E and 6F**).

Together, these data suggest that PINK1 has protective roles against mitochondrial dysfunction and ROS production in renal tubular epithelial cells under high glucose conditions.

### PINK1 inhibits hyperglycemia-associated necroptotic cell death in renal tubular epithelial cells

**Figure 7:**
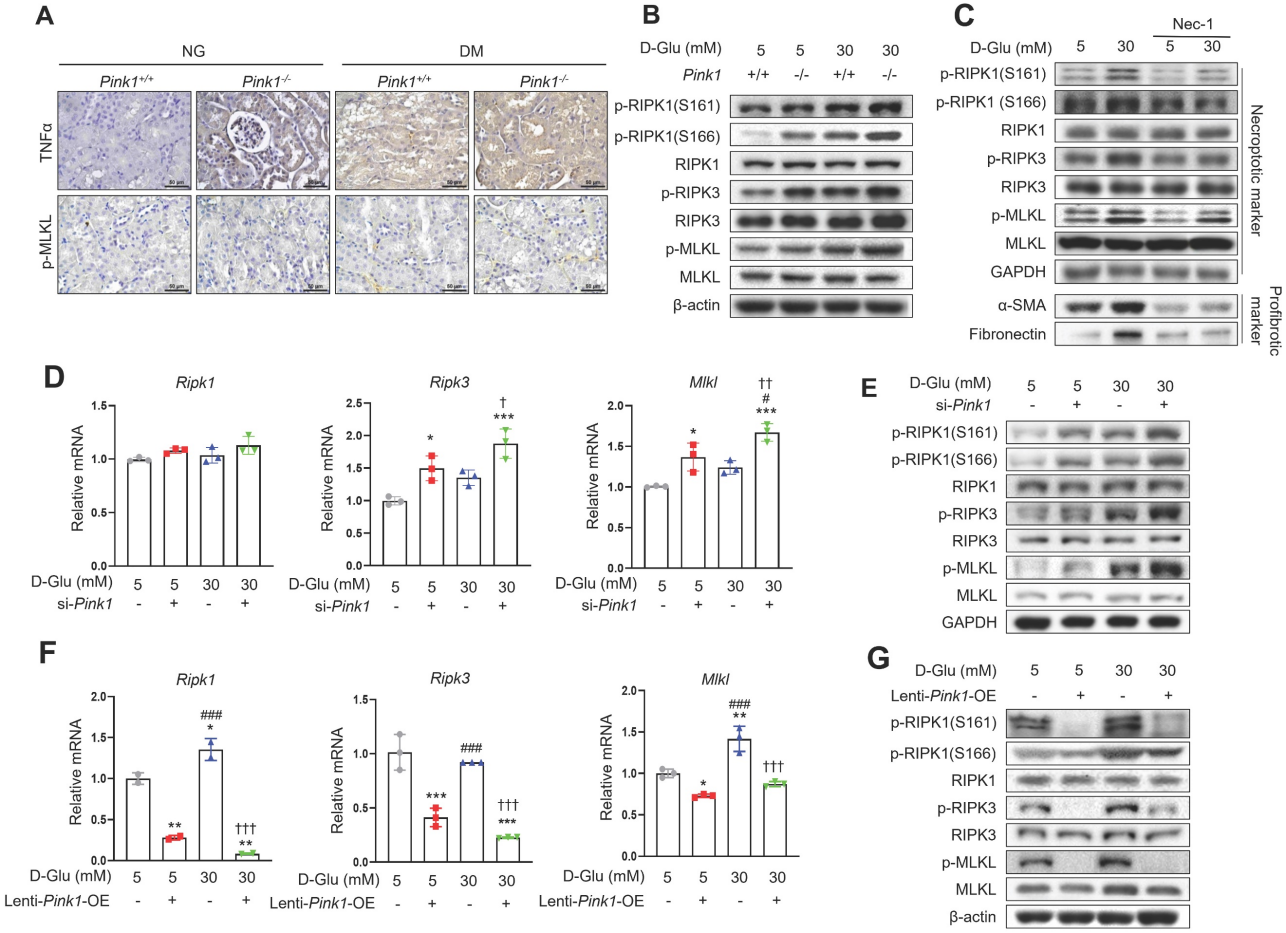
** PINK1 inhibits necroptosis in renal tubular epithelial cells under high glucose conditions. (A)** Representative immunohistochemical images of TNFα and p-MLKL in the kidneys of *Pink1*^+/+^ and *Pink1*^-/-^ with normal or high blood glucose levels. Scale bar = 50 μm. **(B)** Western blotting of necroptosis-related proteins in primary renal tubular epithelial cells of *Pink1^+/+^* and *Pink1^-/-^* mice after treatment with 5- or 30-mM D-glucose. **(C)** Western blotting of necroptosis-related and profibrotic proteins in necrostatin-1-treated HKC-8 cells under 5- or 30-mM D-glucose. **(D)** Quantification of the mRNA expression of necroptosis-related molecules in HKC-8 cells with control or *Pink1* siRNA after treatment with 5- or 30-mM D-glucose. **(E)** Western blotting of necroptosis-related proteins in control or PINK1 siRNA-treated HKC-8 cells under 5- or 30-mM D-glucose.** (F)** Quantification of the mRNA expression of necroptosis-related molecules in control or GFP-*Pink1* infected HKC-8 cells after treatment with 5- or 30-mM D-glucose. **(G)** Western blotting of necroptosis-related proteins in PINK1-overexpressed HKC-8 cells after treatment with 5- or 30-mM D-glucose. Quantitative analysis of **Figure 7B, 7C, 7E, and 7G** are shown in **Figure S3**. (**B-G**) Data are representative of three independent experiments and are expressed as means ± standard error. ^*^p < 0.05, ^**^p < 0.01,^ ***^p < 0.001 vs *Pink1*^+/+^ NG, ^###^*p* < 0.001 vs *Pink1*^-/-^ NG, **^††^***p* < 0.01, **^†††^***p* < 0.001 vs *Pink1*^+/+^ DM. **Abbreviations:** PINK1, PTEN-induced serine/threonine kinase 1; NG, normal glucose; DM, diabetes mellitus; TNFα, tumor necrosis factor α; MLKL, mixed lineage kinase domain-like pseudokinase; RIPK, receptor-interacting protein kinase; α-SMA, alpha-smooth muscle actin; OE, overexpression.

Finally, we explored whether diabetic tubulopathy accelerated by *Pink1* deficiency is implicated in necroptosis. Immunohistochemical staining showed that the expression of TNFα, which is an upstream protein in the necroptosis signaling pathway, and phospho-MLKL, a final component of the necroptosis pathway, were increased by hyperglycemia, and hyperglycemia further increased their expression in the renal tubules (**Figure 7A**). Previous studies demonstrated that RIPK1 phosphorylation at S161 or 166 was involved in the necroptotic signaling pathway initiation 22,26. Particularly, S161, which is an autophosphorylation site of RIPK1, is induced by mitochondrial ROS 22. Primary cultured renal tubular epithelial cells demonstrated that hyperglycemia was associated with increased levels of phospho-RIPK1 at S161 and S166 (**Figure 7B and Figure S3A**). Notably, hyperglycemia-associated elevations in phospho-RIPK1 levels were augmented by PINK1 deficiency. These alterations ultimately increased phospho-MLKL. Necrostatin-1, which is an inhibitor of necroptotic cell death, suppressed the phosphorylation of PINK1 and its downstream proteins and the profibrotic proteins in HKC-8 cells exposed to high glucose media (**Figure 7C and Figure S3B**). *Pink1* siRNA enhanced the expression of necroptosis-related markers (**Figure 7D, 7E, and Figure S3C**), whereas *Pink1* overexpression effectively decreased the expression of key necroptosis components (**Figure 7F, 7G, and Figure S3D**).

Together, our results demonstrate that necroptosis is relevant to diabetic tubulopathy, and importantly, PINK1 restricts necroptosis-related protein expression in tubular epithelial cells under high glucose conditions.

## Discussion

In this study, we investigated the role of PINK1 in DKD. We found that PINK1 deficiency significantly increased albuminuria and aggravated renal tubulointerstitial fibrosis in STZ-induced diabetic mice. Knockdown of *PINK1* promoted profibrotic phenotypes in renal tubular epithelial cells associated with hyperglycemia-induced mitophagic activation suppression and an increase in intracellular ROS, whereas *Pink1* overexpression significantly ameliorated these negative effects. The intracellular stresses caused by PINK1 deficiency ultimately caused enhanced necroptotic responses in renal tubular epithelial cells under hyperglycemic conditions. Together, our data suggest that PINK1 plays an important role in maintaining mitochondrial integrity under hyperglycemic stress; however, its deficiency aggravates DKD through profibrotic transitions and necroptotic cell death of renal proximal epithelial cells.

Kidney fibrosis is a complex pathophysiological process that causes the irreversible accumulation of matrix-producing myofibroblasts. Previous studies consistently demonstrated that proximal tubular injury could contribute to the initiation and progression of kidney fibrosis 27-29. We previously reported that the secretion of profibrotic cytokines in the renal tubular epithelial cells is one of the major pathophysiological processes in diabetic tubulopathy initiation and progression, and the restoration of mitochondrial homeostasis was associated with significant improvements in hyperglycemia-associated kidney fibrosis 16,19,21,30. Here, we demonstrated that impaired mitophagic activity in renal tubular epithelial cells contributes to diabetic tubulopathy aggravation, further highlighting the importance of mitochondrial homeostasis in DKD pathogenesis.

Several previous studies have investigated the clinical relevance of PINK1 in the kidneys under diabetic conditions 8,31-38. However, these studies have produced inconsistent results, possibly attributable to significant variations in experimental designs, diabetes induction methods, sacrifice timings, and the use of different animal models. Moreover, the causality between renal PINK1 expression and sustained hyperglycemia remains uncertain. 8,31,32,35,38. To overcome the limitations of previous research, we conducted our study utilizing PINK1 knockout mice, providing an opportunity to elucidate the pathophysiological roles and cellular mechanisms of PINK1 in DKD. Here, we observed that PINK1 expression significantly increased in renal tubular epithelial cells under hyperglycemic conditions sand that PINK1 deficiency results in defective mitophagic activity and mitochondrial dysfunction, which ultimately worsens hyperglycemia-induced kidney fibrosis. In addition, we successfully established a mechanistic connection between impaired mitophagy and tubular cell necroptosis, a regulated form of cell death not highlighted in DKD. These findings shed light on a novel pathophysiology underlying diabetic tubulopathy and offer potential therapeutic targets for DKD. Notably, PINK1 exerted protective roles in cisplatin-induced acute kidney injury 39, suggesting that PINK1-mediated mitophagic activation represents a common defense mechanism against various types of kidney injuries.

We observed that the loss of *Pink1* was associated with significantly aggravated tubulointerstitial injury and fibrosis in diabetic and in non-diabetic mice (**Figure 1**). These findings suggest that PINK1 plays a role in maintaining kidney homeostasis despite low basal mitophagic activity in renal proximal tubular cells under no-stress conditions 40,41. Therefore, isolated PINK1 deficiency might be sufficient to induce overt kidney injury. Consistently, isolated PINK1 deficiency in HKC-8 cells could perturb mitochondrial homeostasis and induce phenotypic alterations even without hyperglycemic stimuli (**Figure 3-5**).

We examined the effect of PINK1 on the phenotypes of renal tubular epithelial cells using *in vitro* experiments and found that knockdown of *Pink1* increased the expression of profibrotic proteins, including α-SMA, fibronectin, and TGF-β1, under hyperglycemic conditions. Moreover, *Pink1* overexpression significantly attenuated hyperglycemia-induced upregulation of these cytokines, and the restoration of mitochondrial integrity and suppression of ROS production accompanied these beneficial effects. These data indicate that PINK1 is a therapeutic target for treating DKD. Therefore, it is necessary to determine whether drugs that enhance PINK1-mediated mitophagy are also effective in preventing DKD progression.

It is important to address the pathophysiologic roles of PINK1 in mesangial cells and podocytes, as these are critical cell types involved in diabetic glomerulopathy. Previous studies have shown that exposure to hyperglycemic conditions leads to a reduction in PINK1 expression in podocytes, in contrast to the findings in proximal tubular cells as demonstrated in our own data 8,36,37. However, this decrease in PINK1 expression resulted in excessive ROS generation and mitophagic dysfunction, ultimately culminating in podocyte apoptosis. Moreover, the restoration of PINK1 expression was found to reverse the cellular stresses induced by hyperglycemia, highlighting its significance in mitigating these adverse effects on podocytes. Similar to the observations in podocytes, hyperglycemic stimuli were found to downregulate PINK1 expression in mesangial cells, subsequently leading to increased mitochondrial fragmentation and ROS production 42. Collectively, these data emphasize the potential importance of PINK1 in maintaining mitochondrial homeostasis and cell function across different cell types within the diabetic glomerulus.

Programmed cell death is increasingly recognized as a major type of renal tubular cell death and is involved in various renal pathophysiology, including kidney fibrosis 43. Although several studies have demonstrated relevant associations between DKD and ferroptosis, which is another form of regulated necrosis 44-46, , the role of necroptosis in DKD has not been well established. Here, we found that PINK1 deficiency and associated mitochondrial dysfunction were linked to necroptosis in renal tubular epithelial cells. Notably, a link between mitophagy and necroptosis in epithelial cells has also been demonstrated in chronic obstructive pulmonary disease pathogenesis 47.

Interestingly, we observed that hyperglycemia-treated and/or PINK1-deficient HKC-8 cells exhibited increased levels of phospho-RIPK1 at S161 and S166, respectively, both of which were involved in necroptosis initiation 26,48. Particularly, Zhang et al. demonstrated that mitochondrial ROS prosecuted necroptotic cell death through S161 autophosphorylation of RIPK1 48. They also showed that the activated RIPK3 generated intracellular ROS and facilitated necrosome formation, thereby completing the positive feedback loop of ROS and necroptosis 48,49. We speculate that phospho-RIPK at S161 might be primarily responsible for the occurrence of hyperglycemia-induced necroptosis, given the direct associations between hyperglycemia-induced mitophagic dysfunction and mitochondrial ROS (**Figure 5**). However, since necroptosis is reported to be cell-type dependent 50-54, further investigations are required to confirm this hypothesis.

In conclusion, we demonstrated that PINK1 plays a key role in maintaining mitochondrial homeostasis, and that the loss of its function results in DKD aggravation. We also demonstrated that *Pink1* overexpression protected against high glucose-induced mitochondrial dysfunction and necroptosis in renal tubular epithelial cells. These findings might provide novel insights into the mechanisms underlying hyperglycemia-induced renal fibrosis and contribute to developing treatment approaches for DKD. Therefore, further investigations are needed to examine whether pharmacological PINK1 activators can be a novel therapeutic strategy for treating DKD.

## Supplementary Material

Supplementary figures and table.
