# Supplementary material for: PTEN-induced kinase 1 exerts protective effects in diabetic kidney disease by attenuating mitochondrial dysfunction and necroptosis
Source: Int J Biol Sci. 2023 Oct 2;19(16):5145–59. doi: 10.7150/ijbs.83906 (PMC10620829; doi:10.7150/ijbs.83906)
Supplement: Supplementary file 1 — Supplementary figures and table. [file ijbsv19p5145s1.pdf]

SUPPLEMENTAL APPLEDIX FOR THE STUDY:

PTEN-induced kinase 1 exerts protective effects in diabetic kidney disease by attenuating mitochondrial dysfunction and necroptosis

Table S1. Primer sequences used in this study

Gene	Primer sequence
**Human primer**	
**α-SMA**	F: 5'-AAAAGACAGCTACGTGGGTGA-3'<br>R: 5'-GCCATGTTCTATCGGGTACTTC-3'
**Fibronectin**	F: 5'-GCGAGAGTGCCCCTACTACA-3'<br>R: 5'-GTTGGTGAATCGCAGGTCA-3'
**E-cadherin**	F: 5'-CGAGAGCTACACGTTCACGG-3'<br>R: 5'-GGGTGTCGAGGGAAAAATAGG-3'
**TGF-β1**	F: 5'-CAATTCCTGGCGATACCTCAG-3'<br>R: 5'-GCACAACTCCGGTGACATCAA -3'
**PINK1**	F: 5'-CCCAAGCAACTAGCCCCTC-3'<br>R: 5'-GGCAGCACATCAGGGTAGTC-3'
**RIP1**	F: 5'-TGGGCTTCACACAGTCTCAG-3'<br>R: 5'-CCACTTTTGGAGCATCTGGT-3'
**RIP3**	F: 5'-TAGGAAGTGGGGCTACGATG-3'<br>R: 5'-TGGTCCCAGTTCACCTTCTC-3'
**MLKL**	F: 5'-CATCCACAAACGGTGTGAAG-3'<br>R: 5'-GCTCCTCTTTCCTTGGTCCT-3'
**GAPDH**	F: 5'-CGACCACTTTGTCAAGCTCA-3'<br>R: 5'-CCCTGTTGCTGTAGCCAAAT-3'

Gene	Primer sequence
**Mouse primer**	
**α-SMA**	F: 5'-GATGAAGCCCAGAGCAAGAG-3'<br>R: 5'-CTTTTCCATGTCGTCCCAGT-3'
**Fibronectin**	F: 5'-ACAAGGTTCGGGAAGAGGTT-3'<br>R: 5'-CCGTGTAAGGGTCAAAGCAT-3'
**E-cadherin**	F: 5'-TCGGAAGACTCCCGATTCAAA-3'<br>R: 5'-CGGACGAGGAAACTGGTCTC -3'
**TGF-β1**	F: 5'-CCACCTGCAAGACCATCGAC-3'<br>R: 5'-CTGGCGAGCCTTAGTTTGGAC-3'
**PINK1**	F: 5'-TTCTTCCGCCAGTCGGTAG-3'<br>R: 5'-CTGCTTCTCCTCGATCAGCC-3'
**GAPDH**	F: 5'-ACTCCCACTCTTCCACCTTC-3'<br>R: 5'-TCCAGGGTTTCTTACTCCTT-3'

**Primer for human mitochondrial DNA**	
**mtDNA 4,977-bp deletion**	F: 5'-CCTTACACTATTCCTCATCACC-3'<br>R: 5'-TGTGGTCTTTGGAGTAGAAACC-3'
**COX Ⅰ**	F: 5'-TTCGCCGACCGTTGACTATTCTCT-3'<br>R: 5'-AAGATTATTACAAATGCATGGGC-3'
**Nuclear β-actin**	F: 5'-ACCCACACTGTGCCCATCTAC-3'
**Primer for mouse mitochondrial DNA**	
**mtDNA 3,860-bp deletion**	F: 5'-TCATTCTAGCCTCGTACCAACA-3'<br>R: 5'-GAGGTCTGGGTCATTTTCGTTA-3'
**12r**	F: 5'-ACCGCGGTCATACGATTAAC-3'<br>R: 5'-CCCAGTTTGGGTCTTAGCTG-3'

Abbreviation: α-SMA, alpha smooth muscle actin; TGF-β1, transforming growth factor beta 1; PINK1, PTEN-induced kinase 1; RIP, receptor-interacting serine/threonine-protein kinase; MLKL, mixed lineage kinase domain like pseudokinase; GAPDH, glyceraldehyde 3-phosphate dehydrogenase.

Figure S1. Quantitative analysis of western blotting (Figure 2)

A

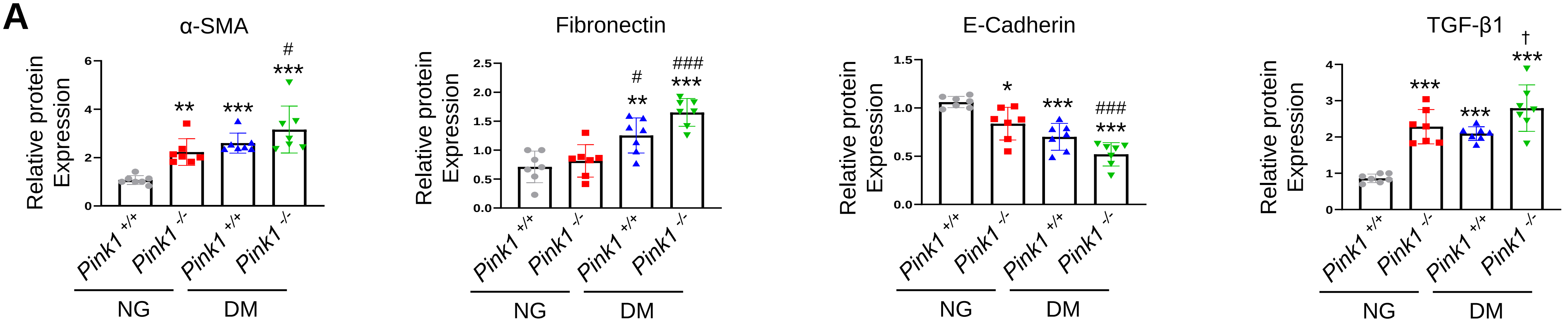

Shown are the quantitative analysis of western blotting in **Figure 2B**.

Data are expressed as means ± standard error. $^{*}p < 0.05$, $^{**}p < 0.01$, $^{***}p < 0.001$ vs *Pink1*$^{+/+}$ NG, $^{\#}p < 0.05$, $^{\#\#\#}p < 0.001$ vs *Pink1*$^{-/-}$ NG, $^{\dagger}p < 0.05$ vs *Pink1*$^{+/+}$ DM

Abbreviations: PINK1, PTEN-induced serine/threonine kinase; α-SMA, alpha-smooth muscle actin; TGF-β1, transforming growth factor-β1; NG, normal glucose; DM, diabetes mellitus.

Figure S2. Quantitative analysis of western blotting (Figure 3)

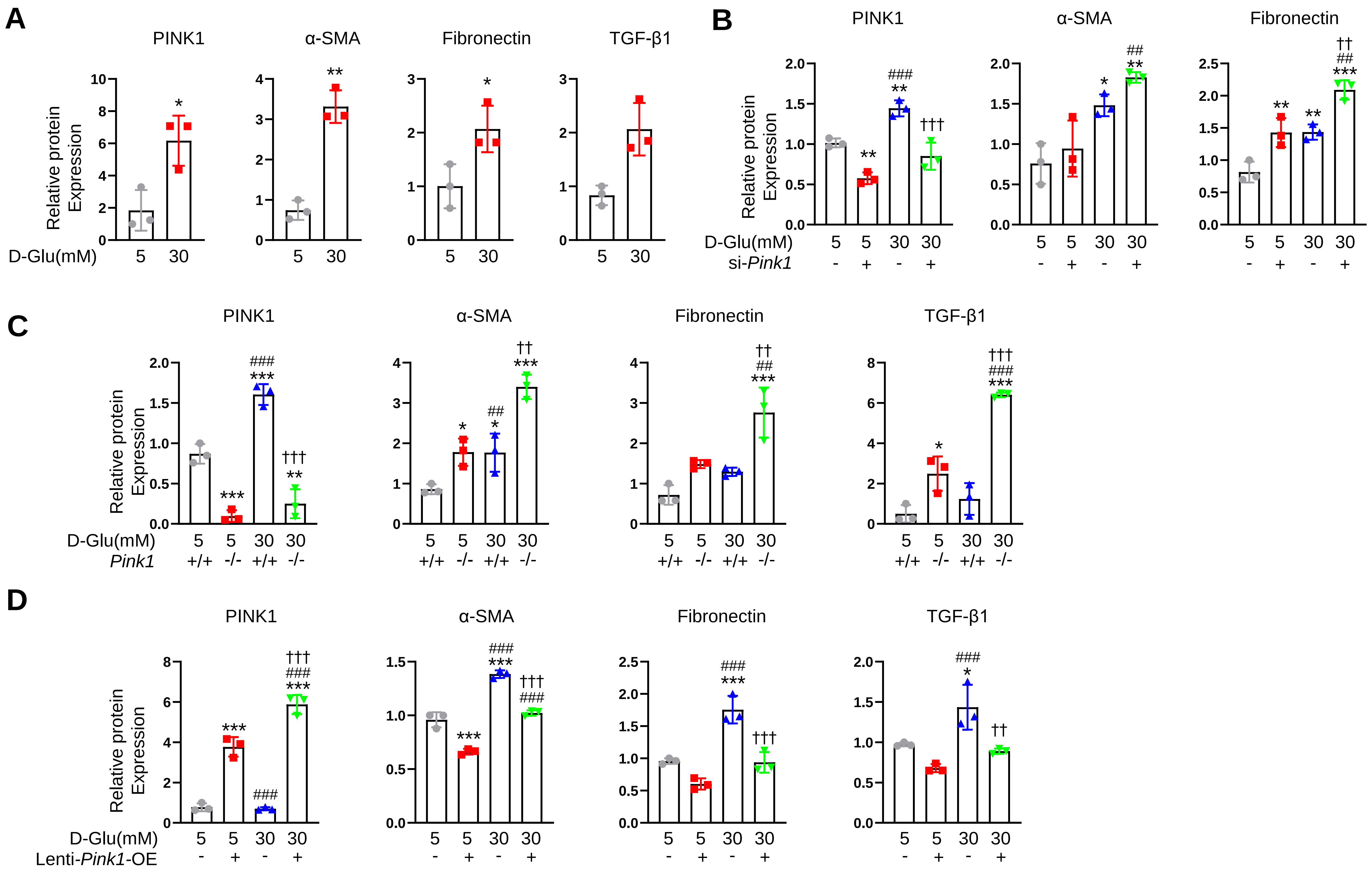

Shown are the quantitative analysis of western blotting in **Figure 3**. Panels **A, B, C**, and **D** correspond to the relative protein expressions of **Figure 3B, 3D, 3F**, and **3H**, respectively.

Data are expressed as means ± standard error. $^{*}p < 0.05$, $^{**}p < 0.01$, $^{***}p < 0.001$ vs *Pink1*$^{+/+}$ NG, $^{\#\#}p < 0.01$, $^{\#\#\#}p < 0.001$ vs *Pink1*$^{-/-}$ NG, $^{\dagger\dagger}p < 0.01$, $^{\dagger\dagger\dagger}p < 0.001$ vs *Pink1*$^{+/+}$ DM

Abbreviations: PINK1, PTEN-induced serine/threonine kinase; α-SMA, alpha-smooth muscle actin; TGF-β1, transforming growth factor-β1; NG, normal glucose; DM, diabetes mellitus; OE, overexpression.

Figure S3. Quantitative analysis of western blotting (Figure 7)

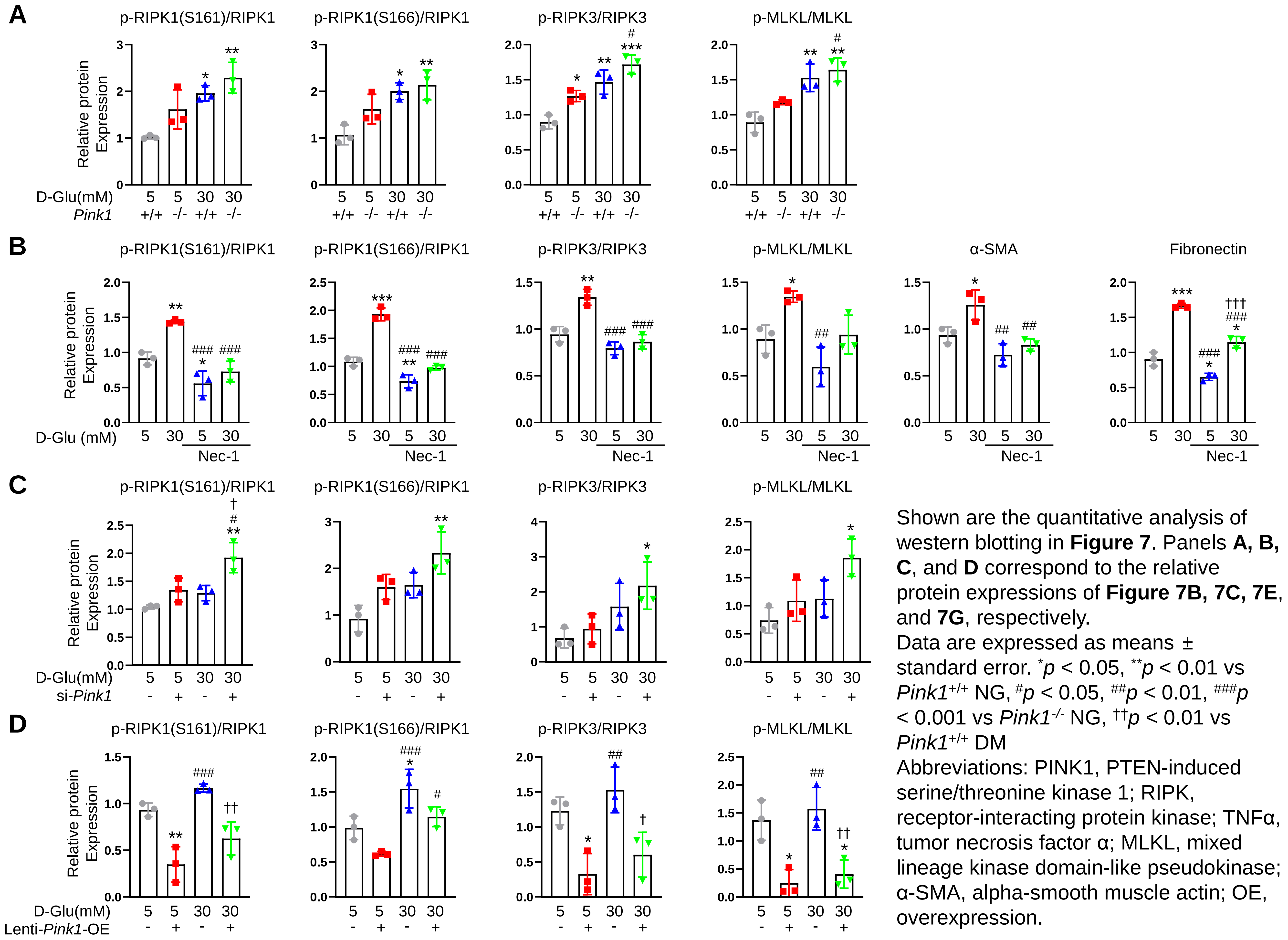

Shown are the quantitative analysis of western blotting in **Figure 7**. Panels **A, B, C**, and **D** correspond to the relative protein expressions of **Figure 7B, 7C, 7E**, and **7G**, respectively.

Data are expressed as means ± standard error. $^{*}p < 0.05$, $^{**}p < 0.01$ vs *Pink1*$^{+/+}$ NG, $^{\#}p < 0.05$, $^{\#\#}p < 0.01$, $^{\#\#\#}p < 0.001$ vs *Pink1*$^{-/-}$ NG, $^{††}p < 0.01$ vs *Pink1*$^{+/+}$ DM

Abbreviations: PINK1, PTEN-induced serine/threonine kinase 1; RIPK, receptor-interacting protein kinase; TNFα, tumor necrosis factor α; MLKL, mixed lineage kinase domain-like pseudokinase; α-SMA, alpha-smooth muscle actin; OE, overexpression.
